# Enhancing the Mechanical Properties of Injectable Nanocomposite Hydrogels by Adding Boronic Acid/Boronate Ester Dynamic Bonds at the Nanoparticle–Polymer Interface

**DOI:** 10.3390/gels10100638

**Published:** 2024-10-02

**Authors:** Jesús Sánchez, Jose Ulloa, Yessenia Oyarzún, Matías Ceballos, Carla Ruiz, Bruno Boury, Bruno F. Urbano

**Affiliations:** 1 Departamento de Polímeros, Facultad de Ciencias Químicas, Universidad de Concepción, Concepción 4030000, Chile; 2 ICGM, CNRS, University Montpellier, ENSCM, 34293 Montpellier, France

**Keywords:** boronic acid, dynamic covalent bonds, nanocomposite hydrogel, viscoelastic properties

## Abstract

Incorporating nanoparticles into injectable hydrogels is a well-known technique for improving the mechanical properties of these materials. However, significant differences in the mechanical properties of the polymer matrix and the nanoparticles can result in localized stress concentrations at the polymer–nanoparticle interface. This situation can lead to problems such as particle–matrix debonding, void formation, and material failure. This work introduces boronic acid/boronate ester dynamic covalent bonds (DCBs) as energy dissipation sites to mitigate stress concentrations at the polymer–nanoparticle interface. Once boronic acid groups were immobilized on the surface of SiO_2_ nanoparticles (SiO_2_-BA) and incorporated into an alginate matrix, the nanocomposite hydrogels exhibited enhanced viscoelastic properties. Compared to unmodified SiO_2_ nanoparticles, introducing SiO_2_ nanoparticles with boronic acid on their surface improved the structural integrity and stability of the hydrogel. In addition, nanoparticle-reinforced hydrogels showed increased stiffness and deformation resistance compared to controls. These properties were dependent on nanoparticle concentration. Injectability tests showed shear-thinning behavior for the modified hydrogels with injection force within clinically acceptable ranges and superior recovery.

## 1. Introduction

Injectable hydrogels have immense potential in various fields, particularly biomedicine, due to their unique properties and versatility [1,2,3]. Their successful use in drug delivery, tissue engineering, and wound healing, as well as their biocompatibility and minimally invasive delivery [4,5], makes them an invaluable tool for advancing healthcare and improving patient outcomes. Despite their advantages, injectable hydrogels also have limitations that need to be addressed, including issues related to their mechanical properties and biodegradation rates. Nanocomposite injectable hydrogels incorporate nanoparticles into the hydrogel matrix to improve the mechanical properties of the hydrogel, add strength and stability, provide additional functionalities, and enhance their overall performance in various applications [6,7]. The strength and mechanical stability of hydrogels can be improved by incorporating nanoparticles. They can also provide responsive behavior to external stimuli such as pH, temperature, and magnetic fields, which are useful for controlled drug release and other therapeutic applications [8,9].

Improving the mechanical properties is associated with increasing the efficiency of transferring stress through the polymer chains to the nanoparticles. Nanoparticles can absorb more mechanical stress since they generally have a larger modulus than the polymer. However, these differences in the rigidity moduli of the two components result in a stress concentration at the nanoparticle–polymer interface [10,11]. These stress concentrations promote the formation of localized cavities at the interface in the first stage, and then large dimples as the stress increases [12,13]. Admittedly, the concentration of nanoparticles has a substantial effect, as more nanoparticles result in more cavitation events and nanoparticle aggregations, among other repercussions. In addition, unless specific treatments are given, the natural and general tendency of the nanoparticles to aggregate promotes the formation of clusters or large filler particles that produce additional energy dissipation mechanisms, such as fracture deflection and fracture pinning [14]. This constitutes another mechanism of mechanical damage in the nanocomposite. Salviato et al. developed a multiscale model (from macroscale to nanoscale) to quantify the energy absorbed by the matrix (*u_m_*) and the interface (*u_a_*) [15]. The model predicts a significant increase in *u_a_* when the radius of the nanoparticles decreases, reaching a contribution of 50% of the total absorbed energy for nanoparticles 10 nm in diameter, thereby demonstrating the importance of the interface in energy dissipation and toughening. Dynamic covalent chemistry (DCC) at the polymer–nanoparticle interface has been shown to significantly improve composite materials’ performance. By incorporating DCC-active moieties at the interface between silica nanoparticles and polymer resins, researchers have demonstrated enhanced stress relaxation, increased toughness, and reduced polymerization shrinkage stress [16]. In dental restorative materials, the implementation of thiol-thioester (TTE) exchange at the resin–filler interface has led to a 30% reduction in shrinkage stress while improving mechanical properties, including a 60% increase in Young’s modulus and a 35% increase in toughness [17].

Further studies on TTE-based adaptive interfaces have revealed a 45% reduction in polymerization stress and significantly increased toughness compared to control composites [18]. These improvements are attributed to the ability of DCC to mitigate interfacial stress concentration and promote stress relaxation, making it a promising approach for enhancing composite performance across various applications. Diodati et al. discovered that incorporating Fe_3_O_4_ nanoparticles into a vinylogous urethane vitrimer did not hinder flow behavior due to their catalytic effect on dynamic exchange chemistry. In addition, the nanoparticles reduced the relaxation time and enabled self-healing properties [19].

This study aims to investigate the polymer–nanoparticle interface in hydrogels by introducing dynamic covalent bonds (DCBs), a type of covalent bond that can break and reform reversibly under certain conditions, such as changes in temperature, pH, or the presence of a catalyst [20]. These DCBs can act as energy dissipation sites through stress relaxation processes (associated with bond exchange) and consequently reduce the interface’s stress concentrations. Usually, the activation of the bond dynamism requires high temperatures or the presence of catalysts [21,22,23]; therefore, not all DBs are suitable to be incorporated into hydrogels since their reversibility does not occur at mild temperatures [24]. Additionally, the unique properties of hydrogels, such as softness, wetness, responsiveness, biocompatibility, and bioactivity, impose some limits on the experimental field conditions needed for their application. This research considers boronic/boronate ester DCBs, reversible condensations of boronic acid and diols that form a cyclic ester occurring under mild conditions, at room temperature, and without a catalyst [25]. The novelty of this research lies in the introduction of boronic acid/boronate ester dynamic covalent bonds at the nanoparticle–polymer interface within injectable nanocomposite hydrogels. Unlike traditional methods, where static covalent bonds at this interface could lead to stress concentrations and potential material failure, the dynamic nature of these bonds allows for stress dissipation.

The formation and stability of boronic acid/boronate ester DCBs are favored at a pH above the pKa of the acid [26]. Under these conditions, the boronate ion with tetrahedral geometry (sp^3^ hybridization) forms a less strained boronate ester ring. Conversely, at pH values below the pKa of the acid, the boronate ester will be less stable and more chemically reversible because of the higher strain of the cyclic ester when it adopts its flat trigonal form (sp^2^ hybridization) [27].

To demonstrate the potential of this type of DCB, we have chosen to work with hydrogels consisting of an alginate matrix reinforced with silica nanoparticles, onto which Wulff-type boronic acid functions have been grafted via organosilane chemistry. Under different pH conditions, the boronic acids can alter their hybridization states and establish dynamic binding interactions at the polymer–nanoparticle interface, resulting in mechanically more stable and injectable hydrogels.

## 2. Results and Discussion

### 2.1. Activation and Functionalization of Nanoparticles

**Figure 1:**
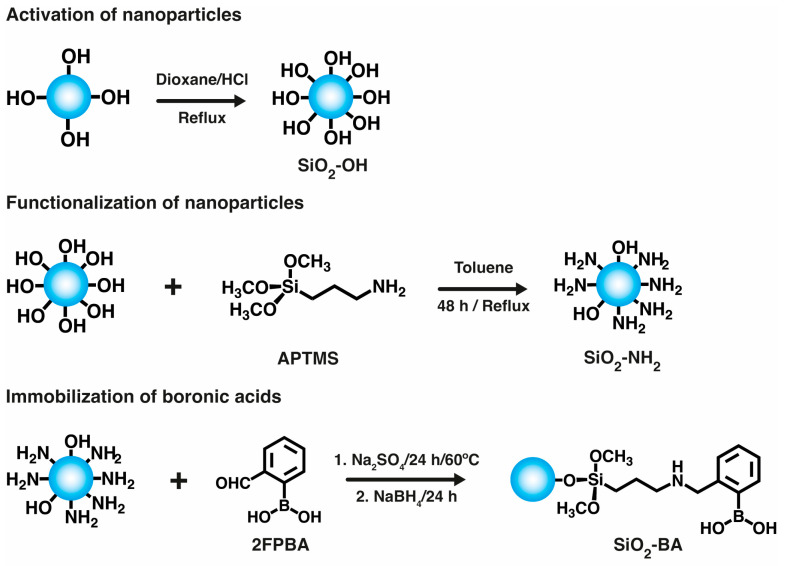
Scheme of activation, functionalization, and immobilization of boronic acid on SiO_2_ nanoparticles.

The strategy for immobilizing boronic acid groups on the surface of the nanoparticles is shown in Figure 1. Commercial SiO_2_ nanoparticles were first activated to increase the density of hydroxyl groups on their surface, resulting in activated SiO_2_-OH nanoparticles [28], which increased the efficiency of the subsequent functionalization process performed with APTMS, resulting in amino-functionalized silica nanoparticles, SiO_2_-NH_2_. The final step to obtain boronic acid-functionalized SiO_2_-BA nanoparticles was a reductive amination reaction between SiO_2_-NH_2_ and 2-formyl phenyl boronic acid (2FPBA). This reaction, catalyzed under acidic conditions, involves the nucleophilic attack of the amino group on the carbonyl group, resulting in the formation of an imine, which is then reduced using sodium borohydride.

**Figure 2:**
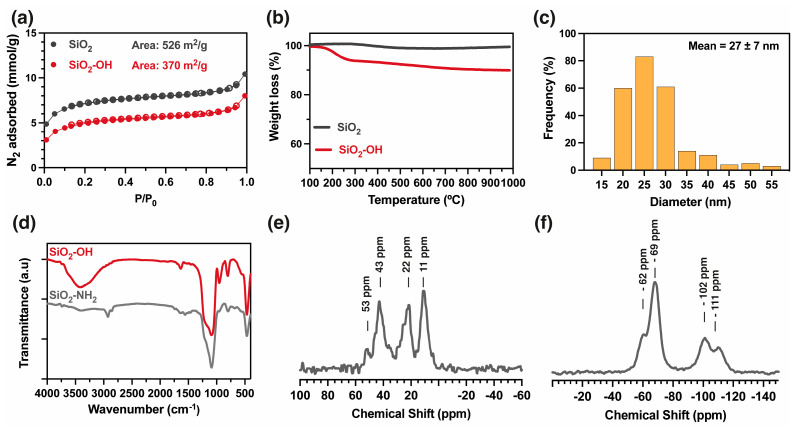
Characterization of nanoparticles. (**a**) N_2_ adsorption isotherms (sorption: close symbols and desorption: open symbols), (**b**) thermogravimetric analyses (10 °C/min, N_2_ atmosphere), (**c**) frequency histogram of nanoparticle diameters (TEM images were analyzed counting at least 250 nanoparticles using software Image J 1.54g), (**d**) FT-IR spectra of activated (SiO_2_-OH) and functionalized (SiO_2_-NH_2_) nanoparticles, (**e**) MAS ^13^C NMR of SiO_2_-NH_2_, (**f**) MAS ^29^Si NMR of SiO_2_-NH_2_.

Figure 2 shows the characterization of the nanoparticles during the functionalization process. Figure 2a shows the N_2_ adsorption isotherms for the initial (SiO_2_) and the activated (SiO_2_-OH) nanoparticles. The adsorption isotherm shows a type II isotherm, indicating macropores in the nanoparticles and inter-nanoparticle voids. The specific surface areas, determined using the Brunauer–Emmett–Teller (BET) method, are 536 and 370 m^2^ g^−1^ for SiO_2_ and SiO_2_-OH, respectively. The thermal decomposition profiles of both samples of nanoparticles show that the SiO_2_ nanoparticles do not exhibit significant mass loss. In contrast, the activated SiO_2_-OH nanoparticles have a 9% mass loss between 180 and 280 °C, which can be attributed to surface dehydroxylation (loss of hydroxyl groups on the surface of the silica) or further condensation of silanol groups (see Figure 2b). The nanoparticle diameter was measured by analyzing TEM images (Figure S1). The frequency histogram shows a diameter range between 20 and 30 nm, with a mean value of 27 ± 7 nm for the SiO_2_-OH nanoparticles (Figure 2c). Subsequently, the activated nanoparticles (SiO_2_-OH) were functionalized with APTMS to obtain SiO_2_-NH_2_, and they were characterized using FT-IR (Figure 2d) along with solid-state ^13^C (Figure 2e) and ^29^Si NMR analyses (Figure 2f). The infrared spectrum of SiO_2_-NH_2_ shows a reduced absorbance at the 3400 cm^−1^ band, implying that the hydroxyl groups interacted with the coupling agent. Additionally, new bands at 2930 cm^−1^ (**ν**, C-H), 1570 cm^−1^ (δ, NH_2_), and 1480 cm^−1^ (**ν**, C-N) indicate the presence of organic moieties on the nanoparticle surface [29]. The ^13^C NMR spectrum reveals signals at 11, 22, and 43 ppm, attributed to the carbons of the propyl moiety, and a signal at 53 ppm, associated with the carbon of the methoxy group of residual Si-O-Me functions. The ^29^Si NMR spectrum of SiO_2_-NH_2_ displays signals centered approximately at Q^3^ (-111 ppm) and Q^4^ (−102 ppm) of SiO_2_; the initial absence of Q^2^ is ascribed to its condensation with APTMS. Furthermore, the spectrum reveals two additional signals at -62 and -69 ppm attributed, respectively, to the silicon atoms of T^1^ (OSi(OH)_2_R) and T^2^ (-O_2_Si(OH)R) units resulting from the condensation of APTMS with Si-OH groups [29]. These findings reveal the successful functionalization of the nanoparticles, but also suggest the persistence of Si-OH functions.

**Figure 3:**
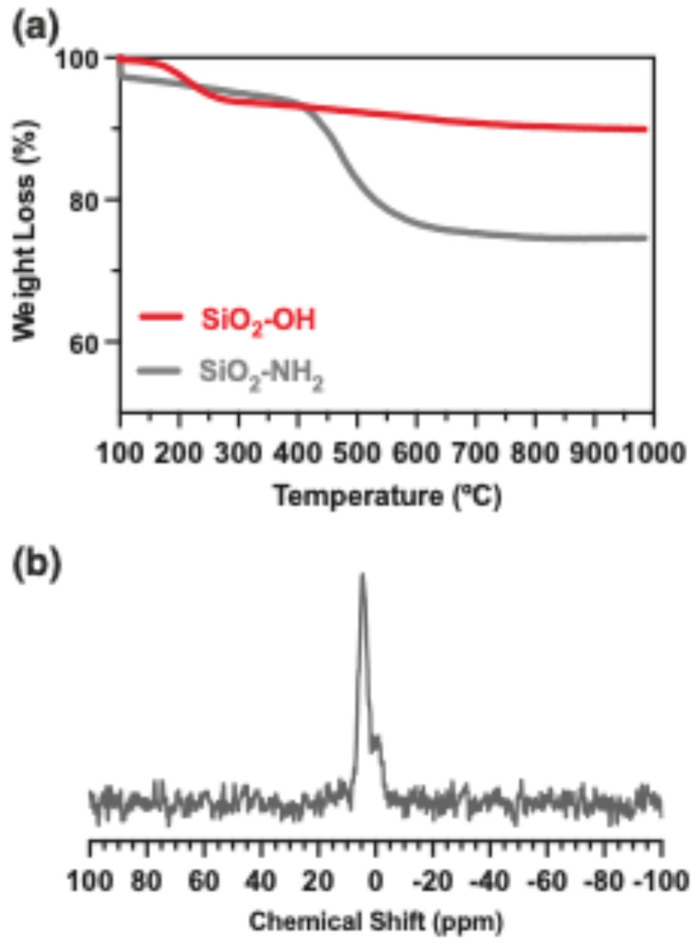
(**a**) TGA of activated and functionalized nanoparticles, (**b**) MAS ^11^B-NMR of immobilized boronic acid on SiO_2_ nanoparticles.

Thermogravimetric analysis was performed to determine the degree of functionalization of the nanoparticles (Figure 3a). Thermal profiles for activated (SiO_2_-OH) and functionalized (SiO_2_-NH_2_) nanoparticles were analyzed. The SiO_2_-NH_2_ nanoparticles show a notable mass loss of 21% between 450 and 600 °C, attributed to the thermal degradation of the organic moiety introduced by APTMS. The difference in mass loss between SiO_2_-OH and SiO_2_-NH_2_ in this range of temperature (450–600) was calculated, along with the size and area of the nanoparticles, with a functionalization degree of 1.63 molecules nm^−2^ [30] (See Supporting Information). In the subsequent step, SiO_2_-NH_2_ nanoparticles were modified with 2-formylphenylboronic acid, yielding SiO_2_-BA. The presence of covalently linked boronic acid on the surface of SiO_2_-BA was confirmed by MAS ^11^B NMR analysis, showing a signal centered at 4.2 ppm that indicates sp^3^ boron hybridization (Figure 3b) [31]. The concentration of boron in SiO_2_-BA was quantified using ICP-MS, and a value of 0.17% was calculated, corresponding to 12% of the amino groups that reacted (see Supporting Information). These data confirm the successful coupling of boronic acid and nanoparticles.

### 2.2. Obtaining the Nanocomposite Hydrogels

**Figure 4:**
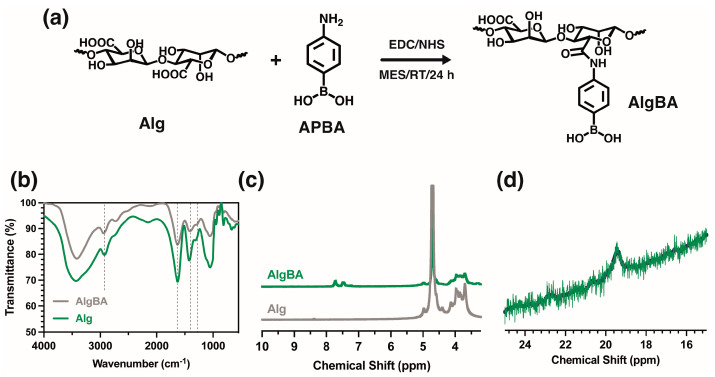
(**a**) Scheme of synthesis of boronic acid-functionalized sodium alginate, and spectroscopic characterizations of (**b**) FTIR, (**c**) ^1^H NMR, (**d**) MASS ^11^B NMR.

The initial goal was to obtain a gel with the nanoparticles in a pure alginate (Alg) matrix, where the boronate esters at the polymer–nanoparticle interface form the hydrogel. In the initial tests involving mixing SiO_2_BA and Alg, a nanoparticle concentration between 0.1 and 2.00 wt% was studied. Evident sedimentation of the nanoparticles was observed at just 1 wt%. A maximum concentration of 0.5 wt% was used to avoid the sedimentation of the nanoparticles. Mixing SiO_2_-BA at 0.5 wt% and unmodified Alg led to the formation of gel (see Figure S1), but it had poor stability over time, attributed to the low concentration of crosslinking sites. For this reason, the alginate was slightly modified with boronic groups to promote gel formation. The modified alginate (AlgBA) was synthesized though a reaction with 4-aminophenyl boronic acid using carbodiimide chemistry to favor the formation of amide bonds between carboxylic and amine groups. The FTIR spectra of sodium alginate and Alg-BA (Figure 4b) show the characteristic bands of the polysaccharides at 3400 cm^−1^ (ν, O-H), 2925 cm^−1^ (ν, C-H), 1638 cm^−1^ (ν as COO-), 1409 cm^−1^ (ν, COO-), 1034 cm^−1^ (δ, O-C-O), and 1300 cm^−1^ (δ, C-C-H) [32]. A difference between the two spectra is the appearance of the bands around 1450 cm^−1^ and 1340 cm^−1^, indicative of the stretching vibrations of C-B and B-O bonds, respectively. In addition, an NMR analysis was performed to confirm the modification of the alginate with the boronic acid derivative. The ^1^H-NMR spectra of both Alg and AlgBA (Figure 4b) show peaks at 7.5 and 8.0 ppm, attributed to the protons associated with the phenyl groups, which are absent in the spectra of the alginate. Other signals between 3.5 and 5.0 ppm, attributed to the protons of the main polymer chain, are not modified by the chemical modification. At the same time, the ^11^B-NMR spectra shown in Figure 4d reveal a peak at 19.37 ppm, attributed to the sp3 hybridization of boron within AlgBA [31], with a concentration of 1% determined by ICP.

**Figure 5:**
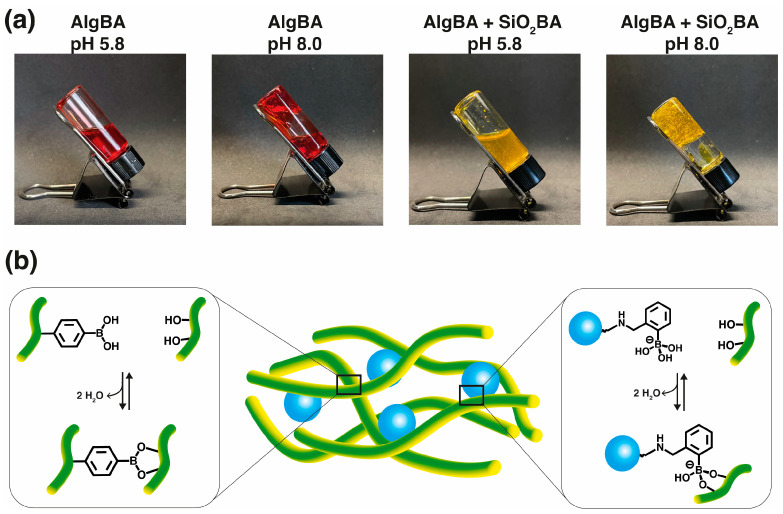
Formation of nanocomposite hydrogel. (**a**) Images of inversion test of AlgBA and AlgBA + SiO_2_BA at two different pHs, forming viscous liquid at pH 5.8 or hydrogels at pH 8.0. (**b**) Illustration of chemical process at work, supporting formation of NCgel by dynamic boronate esters crosslinking at pH 8.0 (pKa 4-aminophenyl boronic acid 8.8, and pKa Wulff-type 5.3 [34]) (alginate in green, silica nanoparticles in blue).

The nanocomposite hydrogels used in this study were prepared using three SiO_2_BA concentrations of 0.1, 0.25, and 0.5 wt% and were named NCgel-0.1, NCgel-0.25, and NCgel-0.5, respectively. AlgBA was used at a concentration of 2.0 wt% in an aqueous solution of 0.1 mol L^−1^ PBS buffer. After dispersing the SiO_2_BA nanoparticles in the aqueous AlgBA solution, the pH was adjusted to 8.0, initiating gelation (see Figure 5a). The gelation is attributed to the formation of dynamic boronic acid/boronate ester covalent bonds between the boronic acid and the vicinal diols of the pyranose rings [33]. However, the structure of the boronic acid is sensitive to changes in pH. Increasing the pH to a number near the pKa of the boronic acid promotes a transition from the neutral form, which has a planar trigonal geometry, to the anionic form, which is characterized by a tetrahedral geometry. Thus, at pH 8.0 and due to the pKa of analogous boronic acids, trigonal esters predominate between the alginate chains. In contrast, the more stable tetrahedral boronate esters predominate at the polymer–nanoparticle interface (see Figure 5b).

The viscoelastic properties of the hydrogels were characterized by rheometry. This technique measures the complex modulus ($G^{*}$), storage modulus ($G^{\prime }$), and loss modulus ($G^{\prime \prime }$). The complex modulus ($G^{*}$) describes the stiffness of the material and summarizes the relationship between the stress and strain responses to applied shear forces. Mathematically, $G^{*}$ is expressed as $G^{*}=\sqrt{{G^{\prime }}^{2}+{G^{\prime \prime }}^{2}\;}$, integrating both the storage modulus ($G^{\prime }$), which refers to the elasticity of the material, and the loss modulus ($G^{\prime \prime }$), which corresponds to the viscous component of the material. In these systems, the temperatures could accelerate the dynamic exchange reactions, leading to changes in the mechanical properties of the gel, so the analyses were limited to a temperature of 37 °C.

**Figure 6:**
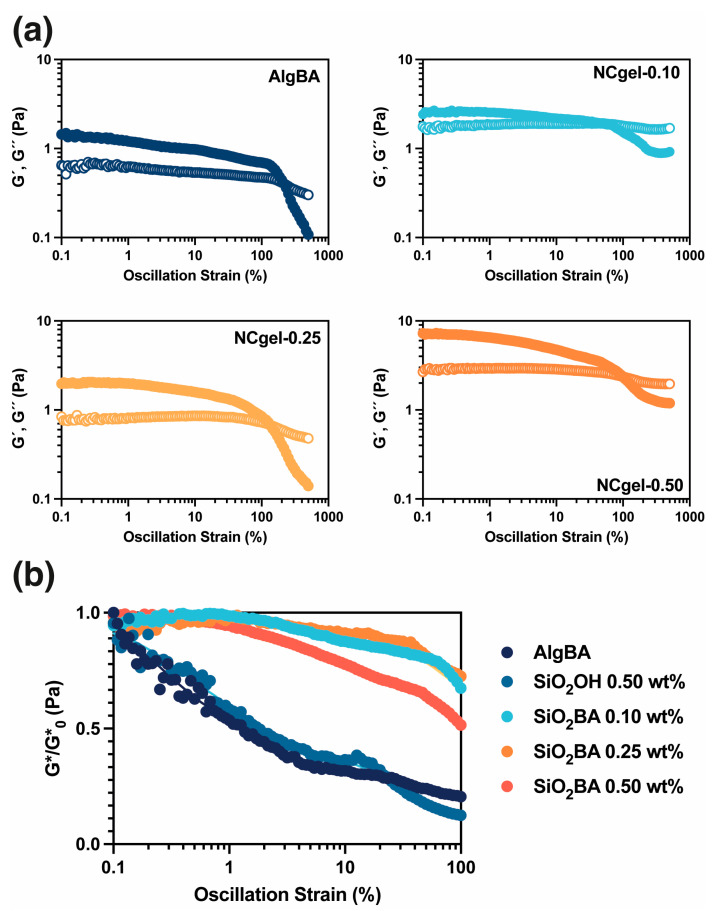
(**a**) Oscillatory strain sweeps of NCgel ($G^{\prime }$ close symbols and $G^{\prime \prime }$ open symbols), (**b**) normalized complex moduli $G^{*}$ of NCgels. Plot (b) includes strain sweeps of NCgel loaded with activated silica (SiO_2_OH) for comparison purposes.

Figure 6a shows the rheological behavior of the samples. All hydrogels showed $G^{\prime }$ > $G^{\prime \prime }$ at reduced strain rates, a characteristic of gel-like properties [35]. In addition, an increase in $G^{\prime }$ was observed for the SiO_2_BA-containing hydrogels, which is theoretically due to the reinforcement by the nanoparticles within the hydrogel matrix. Figure 6b shows the changes in $G^{*}$ with shear strain. A comparison of the normalized $G^{*}$ curves (Figure 6b) show a pronounced decrease in $G^{*}$ AlgBA under minimal strain conditions. Meanwhile, NCgel-0.10 and NCgel-0.25 show superior strain stability. Notably, the NCgels maintain G* over the applied strain spectrum, in contrast to the AlgBA, which experiences an order of magnitude reduction in $G^{*}$, suggesting that incorporating the nanoparticles improves the hydrogel’s stability and deformation resistance. NCgel-0.5 shows greater stability compared to AlgBA, although it is inferior to hydrogels with lower nanoparticle concentrations; this observation could be attributed to nanoparticle aggregation occurring at higher concentrations, with the consequent reduction in nanoparticle distribution within the hydrogel, the formation of areas of elevated nanoparticle concentration, and the decrease in the reinforcing effect. Despite these observations, the exact effect of the nanoparticles, particularly the functionality of the boronic acid groups on their surfaces, on the viscoelastic properties of the hydrogel remained inconclusive. Therefore, a further study was conducted to analyze the performance of a hydrogel loaded only with activated nanoparticles (SiO_2_-OH) at a concentration of 0.5 wt% (Figure 6b). The hydrogels containing activated nanoparticles showed a similar performance to AlgBA. These results support the contention that nanoparticles bearing surface-bound boronic acids enhance the structural integrity and stability of the hydrogel, a phenomenon that can be attributed to energy dissipation mechanisms occurring at the polymer–nanoparticle interface.

### 2.3. Injectability Test

The study of injectable hydrogels plays a central role in various fields, especially regenerative medicine and biomedical applications. Based on reversible chemistry, injectable biomaterials can briefly become fluid under shear stress and then recover their original mechanical properties. From a practical standpoint, the injection force should be of a magnitude that ensures controlled and safe administration. Clinically relevant injection forces are typically less than 20 N [36]. However, the maximum force required to administer an injectable gel can vary considerably, depending on several other factors. These include the rheological properties of the hydrogel, the needle geometry (e.g., diameter), and the specific conditions of the injection procedure, such as injection rate.

**Figure 7:**
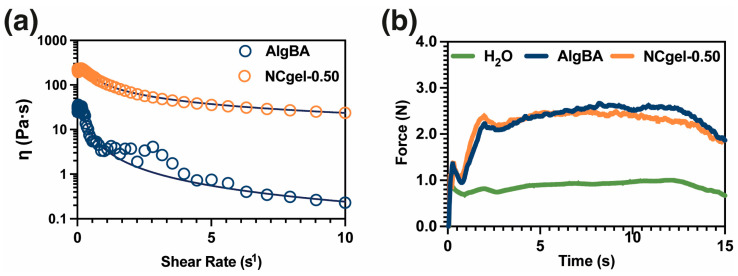
Injectability studies. (**a**) Shear-thinning test [dark line corresponds to Carreau–Yasuda model to determine zero-shear viscosity], (**b**) results of measuring injection force with mechanical testing machine (*n* = 3).

Qualitatively, the hydrogels show the ability to flow smoothly through a syringe and achieve good dimensional stability after injection, allowing control of their injectivity (Videos S1 and S2). The injected hydrogels also exhibit self-repairing properties attributed to dynamic boronic acid-based covalent bonds (Video S3). Various analyses were used to study the injectability of these hydrogels, the first of which was the determination of the shear thinning behavior shown in Figure 7a, characteristic of injectable hydrogels. The presence of SiO2-BA nanoparticles leads to an increase in the zero-shear viscosity value, from 30 to 217 Pa s, and a more subtle reduction in viscosity compared to Alg-BA (Figure 7a and SI videos). In addition, compression tests were performed to determine the injection force. It was observed that both the control and nanocomposite samples exhibit a similar force, approximately 2.10 N, which is within the acceptable limits for clinical applications (Figure 7b).

## 3. Conclusions

This study describes a process to immobilize boronic acid groups on the surface of SiO_2_ nanoparticles, from activation to functionalization, culminating in the successful coupling of boronic acids. The functionalization of silica nanoparticles with boronic acid and the functionalization of sodium alginate with boronic acid are efficient tools for preparing nanocomposite hydrogels with pH-dependent crosslinking. The study of the mechanical properties of these hydrogels has revealed improved viscoelastic properties, attributed to the boronic acid/boronate ester bonds enhanced by the nanoparticles. The rheometric evaluation highlighted the benefits of nanoparticle incorporation, revealing increased hydrogel stiffness and resistance to deformation attributed to the boronic acid at the nanoparticle interface and suggesting a reduced stress concentration at the interface. In terms of practical applications, injectability tests underscored their potential in biomedical applications, as the hydrogels exhibited optimal injection forces of 2.1 N. This research paves the way for advanced nanoparticle-enhanced hydrogel applications.

## 4. Materials and Methods

### 4.1. Materials

All reagents were purchased from Sigma Aldrich (St. Louis, MO, USA) unless otherwise indicated. (2-Formylphenyl)boronic acid (2FPBA, 95%, AK Scientific, Union City, CA, USA), 4-Aminophenylboronic acid hydrochloride (95%, AK Scientific, Union City, USA), Silicon dioxide nanopowder (spherical, porous, 5–20 nm, 99.5%), (3-aminopropyl) trimethoxysilane (APTMS, 97%, 179.3 g/mol), Sodium alginate (Alg), 2-(N-morpholino)ethane sulfonic acid (MES, 99%), Sodium hydroxide (NaOH, 99%), N-hydroxysuccinimide (NHS, 98%), 1-ethyl-3-(3-dimethyl aminopropyl)carbodiimide hydrochloride (EDC·HCl, 98%), triethylamine (TEA), ethanol (98%). 

### 4.2. Activation, Functionalization, and Immobilization of Boronic Acid on SiO_2_ Nanoparticles

First, the nanoparticles underwent activation to increase the concentration of hydroxyl groups on their surfaces. Briefly, 2.0 g of SiO_2_ nanoparticles was dispersed using magnetic stirring in 16.0 mL of 1,4-dioxane in a 50 mL round-bottom flask. The mixture was heated until it reached 80 °C, and 2.0 mL of 3 mol/L HCl was added [28]. The temperature and stirring rate were maintained for 30 min. Subsequently, the solid was separated by centrifugation, washed with the same solvent, and dried at 60 °C overnight in an oven, giving the so-called SiO_2_-OH sample.

Functionalization with amino groups was performed by dispersing 2.0 g of SiO_2_-OH in 50 mL of toluene via magnetic stirring, followed by ultrasonication at 50 Hz for 30 min. After that, APTMS (36.7 mL, 210 mmol) was added to the mixture, followed by heating under reflux for 48 h with constant magnetic stirring, and then the mixture was centrifuged at 4000 rpm for 60 min before being washed with the 1,4-dioxane three times. The final product, SiO_2_-NH_2,_ was obtained after drying overnight at 60 °C in an oven.

In the next step, 1.0 g of SiO_2_-NH_2_, 2FPBA (0.3 g, 2 mmol), and anhydrous sodium sulfate (2.0 g, 14 mmol) were mixed in 100 mL of ethanol and dispersed by ultrasonication at 50 Hz for 10 min, followed by heating to 60 °C and stirring for 24 h. Subsequently, the mixture was cooled in an ice water bath, and sodium borohydride (0.3 g, 8 mmol) was introduced, allowing the reaction to proceed for an additional 24 h at room temperature. The product was then purified through dialysis against water (membrane cut-off 14 kDa) for four days at room temperature. Finally, the nanoparticles featuring boronic acid moieties named SiO_2_-BA were separated by centrifugation at 4000 rpm for 40 min and dried overnight at 60 °C in an oven.

### 4.3. Synthesis of Modified Alginate with 4-Aminophenyl Boronic Acid (AlgBA)

At room temperature, sodium alginate (1.0 g) was dissolved in 100 mL of MES buffer (0.1 mol L^−1^) under constant stirring. The pH was then adjusted to 5.5 by adding NaOH (2 mol L^−1^). Subsequently, EDC (0.7 g, 4 mmol), NHS (0.3 mg, 0.86 mmol), and 4-aminophenyl boronic (0.4 mg, 3 mmol) were added, and the mixture was constantly stirred at room temperature for 24 h. The purification was carried out through dialysis against water (membrane cut-off 14 kDa) for seven days at room temperature, and the solution was finally freeze-dried (lyophilization: −60 °C, 0.054 bar. Telstar, Lyoquest-55) to obtain the dried AlgBA product.

### 4.4. Obtaining the Nanocomposite Hydrogels

The following general procedure was carried out to obtain the nanocomposite hydrogels. In an Eppendorf tube, 1.0 mL of AlgBA dissolution, at a concentration of 2 wt% in phosphate-buffered saline (PBS) 0.1 mol L^−1^, was added. Subsequently, SiO_2_BA nanoparticles (1.0 mg) (Mettler Toledo—XS3DU—Microbalance, OH, USA) were added to the mixture to obtain a final concentration of 0.10 wt%. Avoiding aggregation phenomena to ensure a stable and homogeneous product is crucial. To guarantee this homogeneity, the mixtures were vortexed (Fine Vortex, FINEPCR, Gyeonggi, Korea) before and after pH adjustment to 8.0–8.5 (Thermo Scientific Orion Star A211, Waltham, MA, USA) by adding NaOH aliquots (2 mol L^−1^) to obtain the nanocomposite hydrogels. Two more hydrogels were also prepared with SiO_2_BA concentrations of 0.25 and 0.50 wt%. The nanocomposite hydrogels were named NCgel-x, where x represents the concentration of SiO_2_BA nanoparticles.

### 4.5. Characterization Techniques

The size distribution of the activated nanoparticles was determined utilizing Transmission Electron Microscopy (TEM) (JEM 1200 II, JEOL, Tokyo, Japan). Furthermore, nitrogen adsorption isotherms and the Brunauer–Emmett–Teller (B.E.T) method (TriStar II 3020 V1.03, Micromeritics, Norcross, GA, USA) characterized their specific surface area and isotherm type. Before the measurements, the samples were degassed at 120 °C for 3 h. Alterations in functional groups because of nanoparticle reactions were examined using Fourier-Transform Infrared Spectroscopy (FTIR) (Magna 550 NEXUS, Nicolet, Waltham, MA, USA), within the range of 4000 to 400 cm^−1^. Additional characterization of the nanoparticles was conducted via Nuclear Magnetic Resonance (NMR), which yielded ^1^H, ^11^B, ^13^C, and ^29^Si nuclear magnetic resonance data. The MAS ^11^B solid-state NMR was recorded on a VARIAN VNMRS600 spectrometer at 600 MHz (“Wide Bore” magnet of 14.09 Tesla) equipped with a VARIAN T3 MAS probe. The measurements were carried out using the Single Pulse quantitative technique, with ^1^H decoupling, using a recycling time of 10 s and a π/12 pulse of 1 µs. The samples were run at a speed of 20 kHz. The chemical shift value was calibrated using a secondary reference of NaBH_4_ with a signal of -42.10 ppm. The acquisition window was 100 kHz, and the filtering (line broadening) was 50 Hz. The MAS ^29^Si and ^13^C solid-state NMR were recorded on a VARIAN VNMRS300 spectrometer at 300 MHz (“Wide Bore” magnet of 7.05 Tesla) equipped with a VARIAN T3 MAS probe. The measurements were done using the non-quantitative CPMAS technique with ^1^H decoupling and the quantitative single pulse technique (=one pulse) with ^1^H decoupling. For the CPMAS experiments (non-quantitative), we used a recycling time of 3 s, a π/2 pulse of 5 µs, and a contact time of 2 ms.

Weight loss was measured using a Thermogravimetric Analysis (TGA) instrument (TG 209F1 Iris, Netszch, Selb, Germany) to measure the degree of functionalization. During this process, the samples were housed in alumina capsules under a nitrogen flow rate of 250 mL min^−1^. Initially, they were heated from room temperature to 100 °C at a rate of 10 °C min^−1^ and maintained at this temperature for 30 min, and subsequently, the temperature was increased to 1000 °C at the same heating rate. To quantify the boron content in SiO₂BA and AlgBA, the samples underwent analysis through Inductively Coupled Plasma (ICP) (iCAP RQ ICP-Mass, Thermo Scientific, Waltham, MA, USA). Before this measurement, the samples were submitted to a digestion process using hydrofluoric and nitric acids. The viscoelastic properties of the nanocomposite hydrogels were investigated by rheological analysis (DHR-3, TA Instruments) using parallel plates and a Peltier accessory for temperature regulation. The analysis involved an amplitude sweep ranging from 0.0% to 500%, a plate of 20 mm, a frequency of 1 s^−1^, and a gap of 100 μm, and was carried out in triplicate at a controlled temperature of 37 °C. Shear thinning tests were performed in triplicate, with a shear rate of 0.01 s^−1^ to 10^2^ s^−1^ and a plate of 20 mm, at 37 °C. Time sweeps (120 s) were carried out by applying five cycles of strain, 1% and 1000%, a frequency of 1 s^−1^, and a plate of 20 mm.

Injectability Analysis: This analysis was performed using a texture analyzer (EZ-LX, Shimadzu, Tokyo, Japan) equipped with a 500 N compression load cell. The setup included 1 mL syringes and needles 1.2 mm in diameter (18 gauge) and 38 mm in length. The study was conducted at a 20 mL/min compression speed for 90 s at room temperature, and in triplicate.

## Data Availability

The original contributions presented in the study are included in the article/Supplementary Material, further inquiries can be directed to the corresponding author/s.

## Supplementary Materials

The following supporting information can be downloaded at: https://www.mdpi.com/article/10.3390/gels10100638/s1, Figure S1: Representative TEM images of SiO2-OH nanoparticles; Figure S2: Amplitud sweep of NCgel of SiO2-BA nanoparticles and unmodified alginate, Video S1: Injectability, Video S2: Injectability in dissolution, Video S3: Self-healing properties. https://www.dropbox.com/s/ivqwumxc5by69rt/Movies.zip?dl=0 (accessed on 30 August 2024).
