# Supplementary material for: Enhancing the Mechanical Properties of Injectable Nanocomposite Hydrogels by Adding Boronic Acid/Boronate Ester Dynamic Bonds at the Nanoparticle–Polymer Interface
Source: Gels. 2024 Oct 2;10(10):638. doi: 10.3390/gels10100638 (PMC11507314; doi:10.3390/gels10100638)
Supplement: Supplementary file 1 [file gels-10-00638-s001.zip › gels-3220596-supplementary.pdf]

SUPPLEMENTARY INFORMATION

Enhancing the Mechanical Properties of Injectable Nanocomposite Hydrogels by Adding Boronic Acid/Boronate Ester Dynamic Bonds at the Nanoparticle–Polymer Interface

Jesús Sánchez [1], Jose Ulloa [1], Yessenia Oyarzún [1], Matías Ceballos [1], Carla Ruiz [1], Bruno Boury [2] and Bruno F. Urbano [1,*]

[1] Departamento de Polímeros, Facultad de Ciencias Químicas, Universidad de Concepción, Concepción, 4030000, Chile; jesanchez2019@udec.cl (J.S.); julloa@udec.cl (J.U.); yoyarzun2017@udec.cl (Y.O.); matias.ceballos@emse.fr (M.C.); carlaruiz@udec.cl (C.R.)

[2] ICGM, CNRS, University Montpellier, ENSCM, 34293 Montpellier, France; bruno.boury@umontpellier.fr

* Correspondence: burbano@udec.cl

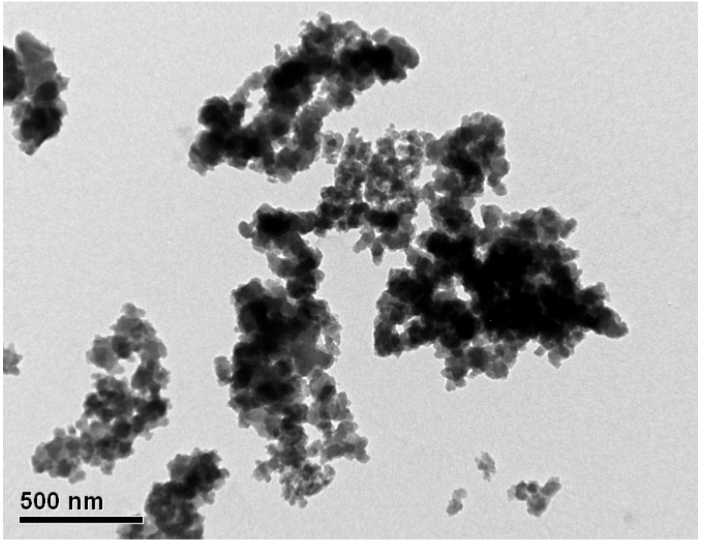

Figure S1. Representative TEM images of SiO_2-OH nanoparticles.

Determination of the grafting degree.

This example is for SiO_2 nanoparticles with a high degree of functionalization according to the method of Kyle C. Bentz and Daniel A. Savin[1]. From the TGA analysis, for SiO_2NH_2, the loss of mass corresponding to organic matter occurs between 385 °C (where there is a residual mass of 96%) and 640 °C (whose residual mass is 80%), assuming that 100 g were burned, the mass corresponding to APTMS (179.3 g/mol) is:

$$\%_{combustion} = \%_{total} - \%_{SiNP} \quad (1)$$

$$\%_{combustion} = 96.0\% - 80.0 = 16.0\% \quad (2)$$

In 100 g of SiO_2NH_2 16.0 g of organic matter is burned, thus the number of silanes grafted is calculated by

$$\left(\frac{16.0\ g\ organic\ fraction}{179.3\ g \cdot mol^{-1}}\right) \cdot N_A = 5.37x10^{22}\ silane\ molecules \quad (3)$$

Where N_A corresponds to Avogadro's number

Similarly, assuming 100 g of total sample, the number of nanoparticles can be found by

$$\frac{\%Si_{TGA}}{m_{SiNP}} = \left(\frac{80.0\ g\ SiO2\ fraction}{1.80x10^{-17}\ g \cdot SiNP^{-1}}\right) = 4.92x10^{18}\ SiNP \quad (4)$$

Where m_{SiNP} corresponds to mass per nanoparticle obtainer by

$$m_{SiNP} = \rho_{SiNP} \cdot V_{SiNP} \quad (5)$$

$$m_{SiNP} = 2.20\ g \cdot cm^{-3} \cdot 8.17x10^{-18} cm^3 = 1.80x10^{-17}\ g \quad (6)$$

Density of SiO_2 is $2.20\ g \cdot cm^{-3}$ and volume of nanoparticle was calculated by

$$V_{SiNP} = \frac{4}{3}\pi\left(\frac{\phi_{TEM}}{2}\right)^3 = 8.17x10^{-18} cm^3 \quad (7)$$

Where ϕ_{TEM} is 25 nm obtained by TEM

The number of silane molecules on each nanoparticle can be found by

$$\frac{5.37x10^{22}\ silane\ molecules}{4.92x10^{18}\ SiNP} = 1.09x10^4\ ligand\ per\ SiO2NP \quad (8)$$

Dividing by the surface areas obtained by BET, we obtain the surface density by

$$grafing\ degree = \frac{1.09x10^4\ ligand \cdot SiO2NP^{-1}}{3.70x10^{20} nm \cdot g^{-1} \cdot 1.80x10^{-17}\ g} \quad (9)$$

$$grafing\ degree = 1.63\ ligand \cdot nm^{-2} \quad (10)$$

Determination of the percentage of functionalization with boronic acid.

Asuming that the area of SiO_2-NH_2 is the same of activated nanoparticles (SiO_2-OH), then

$$grafing\ degree = \frac{1.63\ molec \cdot nm^{-2}\ x\ 526\ m^2 \cdot g\ NP^{-1}}{10^{-18} m^2 \cdot nm^{-2} x\ 6.02x10^{23} molec \cdot mol^{-1}} \quad (11)$$

$$grafing\ degree = 1.42x10^{-3} mol\ NH_2 \cdot g\ NP^{-1} \quad (12)$$

The boron concentration is 0.17%, then

$$B\ grafting = \frac{1.7x10^{-3} g\ B \cdot g\ NP^{-1}}{10.11\ g\ B \cdot mol^{-1}} \quad (13)$$

$$B\ grafing = 1.68x10^{-4} mol\ B \cdot g\ NP^{-1} \quad (14)$$

if 1 mol boronic acid react with 1 mol de amino groups, then $1.68x10^{-4} mol\ B$ reacts with $1.68x10^{-4} mol\ NH_2$

$$Amino\ reacted = \frac{1.68x10^{-4} mol\ NH_2}{1.42x10^{-3} mol\ NH_2} x100 \quad (15)$$

$$Amino\ reacted = 12\ \% \quad (16)$$

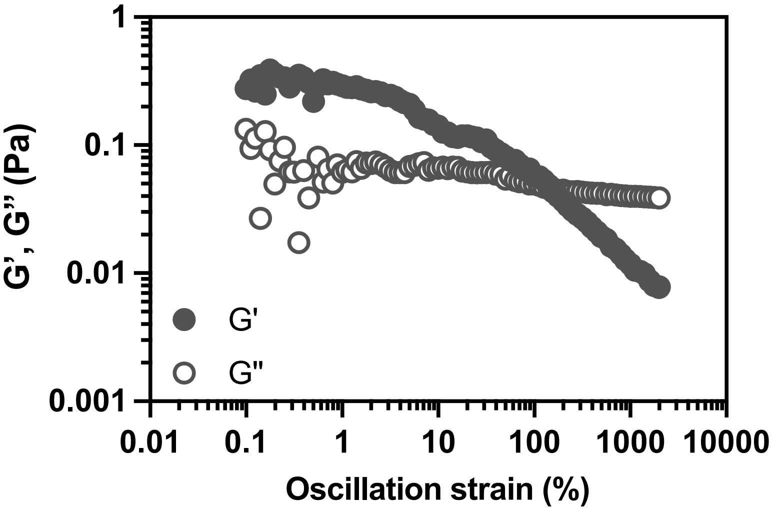

Figure S2. Amplitud sweep of NCgel of SiO_2-BA nanoparticles and unmodified alginate.

Video S1: Injectability, Video S2: Injectability in dissolution, Video S3: Self-healing properties. https://www.dropbox.com/s/ivqwumxc5by69rt/Movies.zip?dl=0

References

1. Bentz, K. C.; Savin, D. A. Chain Dispersity Effects on Brush Properties of Surface-Grafted Polycaprolactone-Modified Silica Nanoparticles: Unique Scaling Behavior in the Concentrated Polymer Brush Regime. *Macromolecules* **2017**, *50*, 5565-5573.
